# Interrupted Time-Series Analysis of Regulations to Reduce Paracetamol (Acetaminophen) Poisoning

**DOI:** 10.1371/journal.pmed.0040105

**Published:** 2007-04-03

**Authors:** Oliver W Morgan, Clare Griffiths, Azeem Majeed

**Affiliations:** 1 Department of Primary Care and Social Medicine, Imperial College London, United Kingdom; 2 Mortality Statistics Branch, Office for National Statistics, United Kingdom; University College London, United Kingdom

## Abstract

**Background:**

Paracetamol (acetaminophen) poisoning is the leading cause of acute liver failure in Great Britain and the United States. Successful interventions to reduced harm from paracetamol poisoning are needed. To achieve this, the government of the United Kingdom introduced legislation in 1998 limiting the pack size of paracetamol sold in shops. Several studies have reported recent decreases in fatal poisonings involving paracetamol. We use interrupted time-series analysis to evaluate whether the recent fall in the number of paracetamol deaths is different to trends in fatal poisoning involving aspirin, paracetamol compounds, antidepressants, or nondrug poisoning suicide.

**Methods and Findings:**

We calculated directly age-standardised mortality rates for paracetamol poisoning in England and Wales from 1993 to 2004. We used an ordinary least-squares regression model divided into pre- and postintervention segments at 1999. The model included a term for autocorrelation within the time series. We tested for changes in the level and slope between the pre- and postintervention segments. To assess whether observed changes in the time series were unique to paracetamol, we compared against poisoning deaths involving compound paracetamol (not covered by the regulations), aspirin, antidepressants, and nonpoisoning suicide deaths. We did this comparison by calculating a ratio of each comparison series with paracetamol and applying a segmented regression model to the ratios. No change in the ratio level or slope indicated no difference compared to the control series. There were about 2,200 deaths involving paracetamol. The age-standardised mortality rate rose from 8.1 per million in 1993 to 8.8 per million in 1997, subsequently falling to about 5.3 per million in 2004. After the regulations were introduced, deaths dropped by 2.69 per million (*p* = 0.003). Trends in the age-standardised mortality rate for paracetamol compounds, aspirin, and antidepressants were broadly similar to paracetamol, increasing until 1997 and then declining. Nondrug poisoning suicide also declined during the study period, but was highest in 1993. The segmented regression models showed that the age-standardised mortality rate for compound paracetamol dropped less after the regulations (*p* = 0.012) but declined more rapidly afterward (*p* = 0.031). However, age-standardised rates for aspirin and antidepressants fell in a similar way to paracetamol after the regulations. Nondrug poisoning suicide declined at a similar rate to paracetamol after the regulations were introduced.

**Conclusions:**

Introduction of regulations to limit availability of paracetamol coincided with a decrease in paracetamol-poisoning mortality. However, fatal poisoning involving aspirin, antidepressants, and to a lesser degree, paracetamol compounds, also showed similar trends. This raises the question whether the decline in paracetamol deaths was due to the regulations or was part of a wider trend in decreasing drug-poisoning mortality. We found little evidence to support the hypothesis that the 1998 regulations limiting pack size resulted in a greater reduction in poisoning deaths involving paracetamol than occurred for other drugs or nondrug poisoning suicide.

## Abstract 2

Analysis of mortality rates for paracetamol poisoning in England and Wales does not support the view that regulations limiting pack size have been responsible for a reduction in deaths.

## Editors' Summary

**Background.**

Paracetamol—known as acetaminophen in the United States—is a cheap and effective painkiller. It is widely used to relieve minor aches and pains as well as fevers and headaches. Recommended doses of paracetamol are considered safe in humans, but overdoses are toxic and can cause liver failure and death. Because this drug is very easy to get hold of, there are many overdoses each year, either accidental or deliberate. In the UK, paracetamol poisoning is the most common cause of acute liver failure. Toward the end of 1998, new laws were introduced in the UK to try to reduce the number of paracetamol overdoses. These laws said that pharmacies could not sell packs of paracetamol containing more than 32 tablets and other shops could not sell packs with more than 16 tablets. One of the reasons behind the introduction of this law was that many suicides are not preplanned and, therefore, if it was harder for people to get hold of or keep large quantities of tablets, they might be less likely to attempt suicide or accidentally overdose.

**Why Was This Study Done?**

Following the introduction of these new laws, the number of deaths caused by paracetamol overdose in the UK dropped. However, it is possible that the drop in deaths came about for a variety of different reasons and not just as a result of the new laws on paracetamol pack size. For example, the suicide rate might have been falling anyway due to other changes in society and the fall in death rate from paracetamol might just have been part of that trend. It is important to find out whether the legal changes that were introduced to address a public health problem did in fact bring about a change for the better. This knowledge would also be relevant to other countries that are considering similar changes.

**What Did the Researchers Do and Find?**

The researchers used data from the Office of National Statistics, which holds information on drug poisoning deaths in England and Wales. These data were then broken down by the type of drug that was mentioned on the death certificate. The researchers compared death rates involving the following drugs: paracetamol; paracetamol-containing compounds (which were not subject to the new pack size laws); aspirin; antidepressant drugs; and then finally non-drug poisoning suicides. The reason for comparing death rates involving paracetamol against death rates involving other drugs, or non-drug suicide, was that this method would allow the researchers to see if the drop in paracetamol deaths followed overall trends in the poisoning or suicide rates or not. If the paracetamol death rate dropped following introduction of the new laws but the rates of other types of poisoning or suicide did not, then there would be a link between the new laws and a fall in paracetamol suicides. The researchers compared these death data within specific time periods before the end of 1998 (when the new laws on paracetamol pack size were introduced) and after.

Overall, there were nearly 2,200 deaths involving paracetamol between 1993 and 2004. The number of deaths per year involving paracetamol dropped substantially when comparing the periods of time before the end of 1998 and after it. However, the number of deaths per year involving any drug, and the non-drug suicides, also fell during this period of time. When comparing the trends for paracetamol deaths with other poisoning or suicide deaths, the researchers did not find any statistical evidence that the fall in paracetamol deaths was any different to the overall trend in poisoning or suicide death rates.

**What Do These Findings Mean?**

Although the paracetamol death rate fell immediately following the new laws on pack size, this study suggests the link might just be coincidence. The researchers could not find any data supporting the idea that the new laws caused a drop in paracetamol deaths. However, this was an observational study, not a true experimental one: the researchers here were clearly not able to set up equivalent “experimental” and “control” groups for comparison. It is very difficult to prove or disprove conclusively that new laws such as this are, or are not, effective.

**Additional Information.**

Please access these Web sites via the online version of this summary at http://dx.doi.org/10.1371/journal.pmed.0040105

- Information is available from Medline Plus about suicide
- Wikipedia has an entry on paracetamol (note that Wikipedia is an internet encyclopedia anyone can edit)
- Information about regulation of drugs in the UK is available from the Medicines and Healthcare Regulatory Agency
- The Office for National Statistics provides key economic and social data about the UK, and is involved in many other important projects

## Introduction

Paracetamol (acetaminophen) is an effective, cheap, and widely available analgesic. However, poisoning due to paracetamol is a common problem worldwide [1] and is currently the most common cause of acute liver failure in UK [2] and the United States [3]. Each year in England and Wales, there are approximately 150 deaths and over 30,000 hospital admissions due to paracetamol overdose [4]. Successful interventions to reduce harm from paracetamol poisoning are needed.

In September 1998, the Medicines Control Agency of the United Kingdom introduced legislation to limit the availability of paracetamol and thereby reduce mortality and morbidity due to paracetamol poisoning [5,6]. Availability of aspirin was similarly limited to avoid substitution. Pack sizes were limited to 16 tablets at general sales outlets and 32 tablets at pharmacies. The maximum number of tablets that can be sold in any single purchase without a prescription was limited to 100 tablets. Concurrently, the government agreed voluntary restrictions with the pharmaceutical industry and retailers to sell no more than 32 tablets in any single purchase.

Several studies have shown that paracetamol poisoning deaths in England and Wales have decreased, although they are unable to convincingly attribute this to the 1998 regulations [7–9]. Here we use interrupted time-series analysis to assess whether the recent fall in the number of paracetamol deaths is different to trends in fatal poisoning involving aspirin, paracetamol compounds, antidepressants, or nondrug poisoning suicide.

## Methods

### Mortality Data

The Office for National Statistics (ONS) maintains a dedicated database of drug-poisoning deaths in England and Wales since 1993. Drug-poisoning deaths are extracted from the national deaths database using specific International Classification of Diseases codes for the underlying cause of death [10]. In addition to data supplied in the cause of death section of the coroner's death certificate, the database also contains textual information supplied voluntarily and in confidence by coroners to ONS about circumstances of the death, which may include more detailed information from an inquest investigation about the drugs involved [10]. Two trained ONS coders independently code the textual information about all drugs mentioned by the coroner. Any discrepancies are resolved and discussed with a senior medical epidemiologist. The manual coding allows the database to be queried and all deaths identified where specific drugs were detected by the coroner. About 90% of drug-poisoning deaths have specific information about the drug(s) taken [4].

We identified deaths in which paracetamol was mentioned on the death certificate, with or without alcohol or other nonparacetamol drugs. Where death certificates mentioned paracetamol along with codeine, dihydrocodeine, dextropropoxyphene, or as part of a paracetamol-containing drug (e.g., Paramax and Propain), these were defined as being paracetamol compound-related deaths. To ascertain whether changes in mortality trends for paracetamol poisoning were coincidental rather than due to the intervention, we compared paracetamol deaths with paracetamol compounds, aspirin, antidepressant drugs, and suicide deaths involving methods other than drug poisoning [11]. We chose antidepressants because along with paracetamol, they are the most common substances mentioned in drug-related poisoning suicides [11]. Therefore, if changes in the time series for paracetamol were due to overall changes in the propensity to commit suicide by drug overdose rather than the regulations, we would expect to see similar changes for antidepressants. Furthermore, analysing nondrug poisoning suicides should give an indication of any effect of suicide trends overall. We did not compare deaths involving paracetamol with those involving opiates, even though they make up about half of all drug-poisoning deaths [4]. This was because the epidemiology of opiate-related deaths differs markedly from other drug-related deaths, having increased more than 6-fold between 1993 and 2000 [12].

### Analysis

We calculated annual directly age-standardised mortality rates using the European Standard population. For the interrupted time-series analysis, we used segmented linear regression, which divides a time series into pre- and postintervention segments [13]. As the regulations were introduced at the end of 1998, we chose 1999 as the intersection between segments (i.e., the intervention). A linear regression model has two parameters: the level and slope. Therefore the difference between the two segments can be quantified by testing the change in these two parameters (Equation 1). A change in level between the pre- and postintervention segments indicates a step-change, and a change in slope indicates a change in trend.

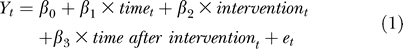



*β*_0_ estimates the baseline level of the outcome at the beginning of the time series. *β*_1_ estimates the preintervention trend where *time* is a continuous variable indicating the time in years at time t from the start of the study period. *β*_2_ estimates the change in level postintervention where *intervention*_t_ = 0 before the intervention, and *intervention*_t_ = 1 after the intervention. *β*_3_ estimates the change in postintervention trend where *time after intervention* is a continuous variable indicating the number of years after the start of the intervention at time t and is coded as zero before the intervention. *e*_t_ includes random error and autocorrelation.

Time-series data are often autocorrelated (events closer together in a time series tend to be more similar than events further apart in time) [14]. Hence, the model residuals are not independent (a key assumption when using ordinary least-squares regression) [15]. Nonindependence of the residuals can lead to biased standard deviations, which can over- or underestimate tests of statistical significance [16]. We therefore corrected for autocorrelation effects by including a term in the regression model for the lagged residuals (i.e., the residual of the regression model moved to the previous time points in the time series).

We considered whether changes in level and slope for paracetamol poisoning were different from the comparison series (compound paracetamol, aspirin, antidepressants, and nondrug poisoning suicide). We made this comparison by calculating a ratio for each year by dividing the age-standardised mortality rates for each comparison group by paracetamol and used segmented regression to assess changes in the level and slope of the ratios, again using 1999 as the intersection between segments. The null hypothesis was that changes in the age-standardised mortality rates for paracetamol and the comparison groups were similar. We rejected the null hypothesis if there was statistical evidence that the model parameters (level or slope) of the ratios were statistically different from 0 at the *p* < 0.05 level between the pre- and postintervention segments. The 95% confidence intervals of the ratio of the model parameters summarize the degree of difference between paracetamol and the control series that can be excluded as not being statistically different. All analysis was done using Stata 8.2 (Stata, http://www.stata.com).

## Results

**Figure 1:**
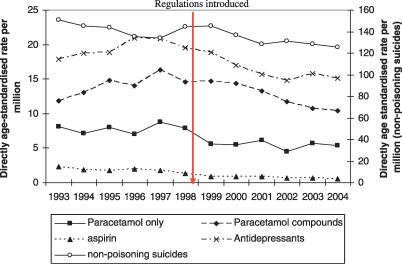
Directly Age-Standardised Mortality Rates per Million, England and Wales, 1993–2004

Between 1993 and 2004, 2,196 poisoning deaths occurred involving paracetamol in England and Wales. Number of deaths were similar among males and females, and hence we present results for males and females combined. Changes in age-specific mortality rates were similar across all age groups during the study period (unpublished data). For paracetamol poisoning the age-standardised mortality rate was 8.1 per million in 1993, peaking at 8.8 per million in 1997, followed by a decrease to 5.3 per million in 2004 (Figure 1; Table 1).

**Table 1:**
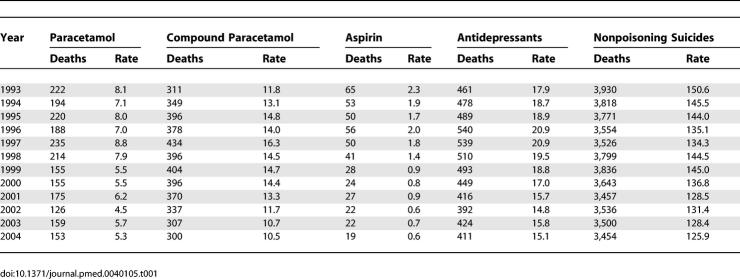
Number of Deaths and Age-Standardised Mortality Rate per Million for Poisoning Involving Paracetamol, Paracetamol Compounds, Antidepressants, and Nondrug Poisoning Suicide, England and Wales

The results of the segmented regression analysis for paracetamol poisoning are summarised in Table 2. There was evidence of first-order autocorrelation (*p* = 0.033), and so we included a term for it in the final model. There was a downward step-change in the annual age-standardised mortality rate, of −2.69 per million (*p* = 0.003) between the pre- and postintervention periods, while there was no evidence of a change in slope (*p* = 0.128) in the postintervention period.

**Table 2:**
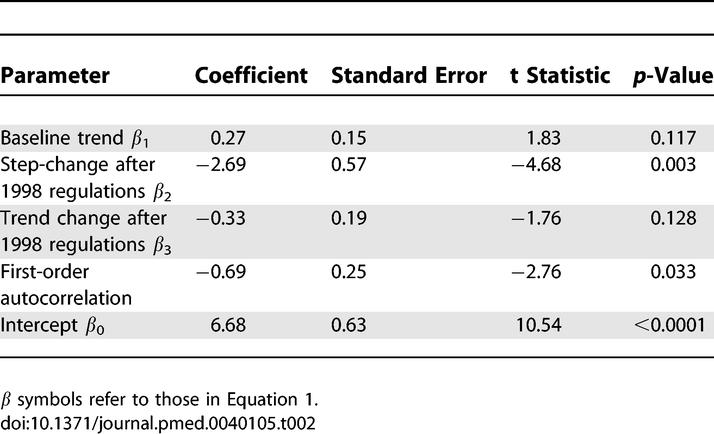
Interrupted Time-Series Regression Analysis of Age-Standardised Mortality Rates for Paracetamol Poisoning, Adjusted for First-Order Autocorrelation

About twice as many deaths involved paracetamol compounds (*n* = 4,378), less than one-fourth of deaths involving aspirin (*n* = 457), two and a half times as many involving antidepressants (*n* = 5,602), and almost 20 times as many deaths due to nondrug poisoning suicide (*n* = 43,824) (Table 3). The median age at death for aspirin, 62 years, was higher than other groups (Table 3). A similar proportion of deaths involved males and females for all three drug-poisoning groups, but not nondrug suicide deaths, for which about 80% were male. Compared to paracetamol compounds and antidepressants, paracetamol deaths were less likely to mention other drugs, whereas aspirin deaths were more likely to mention other drugs. The majority of deaths were suicides for all groups except aspirin, among which only about half were considered suicide.

**Table 3:**
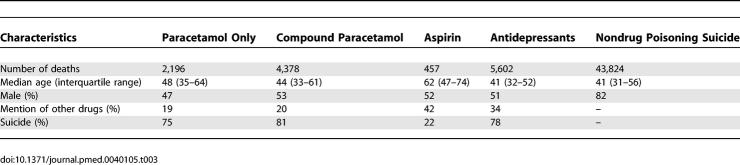
Number of Deaths, Median Age, and Percentages: Male, Mentions of Other Drugs, and Suicides, England and Wales 1993–2004

Age-standardised mortality rates for paracetamol compounds, aspirin, antidepressants, and nonpoisoning suicides all declined over the study period (Figure 1; Table 1). In the preintervention period, rates for paracetamol compounds and antidepressants increased slightly, whereas paracetamol-poisoning deaths and nondrug poisoning suicides were relatively stable (Figure 1). In the postintervention period, rates for all series declined, with paracetamol compounds declining the most.

A summary of the segmented regression models applied to the ratios of the comparison with paracetamol series is shown in Table 4. The time series for paracetamol compounds differed from paracetamol, having a greater step-change (coefficient = 0.81; *p* = 0.012) and greater postintervention decline (coefficient = −0.19; *p* = 0.031). In contrast, no statistical evidence suggested that the change in trends for aspirin or antidepressants was different to the change in paracetamol trends. The ratio of aspirin versus paracetamol showed a similar postintervention step-change (coefficient = 0.01; *p* < 0.779) and slope (coefficient = −0.003; *p* = 0.834). Likewise, antidepressants had a similar step-change (*p* = 0.108) and change in slope (*p* = 0.250). Nondrug poisoning suicide, on the other hand, showed a statistically marked upward step-change (coefficient = 8.44; *p* = 0.025) but a similar postintervention trend (*p* = 0.865). The upward step-change in the ratio of paracetamol versus nondrug poisoning suicide was due to a fall in paracetamol deaths. Examination of the number of deaths in Table 1 and the age-standardised rates in Figure 1 shows that only a small increase occurred in nondrug poisoning suicide between 1998 and 2000, which was within the bounds of measurement error and random annual variation.

**Table 4:**
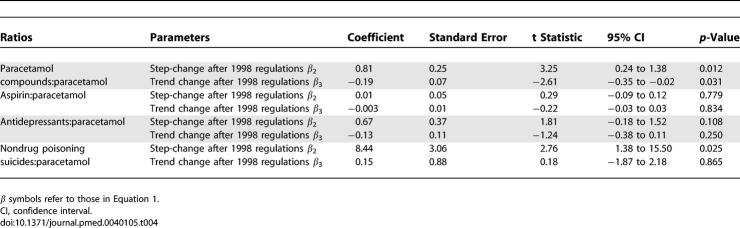
Interrupted Time-Series Regression Analysis of the Ratio of Age-Standardised Poisoning Mortality Rates for Paracetamol Compounds, Antidepressants, and Nondrug Poisoning Suicide:Paracetamol

## Discussion

Between 1993 and 2004, there was a decline in age-standardised mortality rates from paracetamol poisoning. The decline did not occur gradually over the study period, but there was a downward step-change coinciding with the introduction of regulations to limit availability of paracetamol. This step-change was similar for poisoning deaths involving antidepressants and aspirin, but not paracetamol compounds or nondrug poisoning suicide. The 95% confidence intervals of the coefficients for aspirin and antidepressants show that statistical power was sufficient to detect a relatively small divergence in the ratios with paracetamol, thereby increasing our confidence in the conclusion that trends were similar to paracetamol poisoning.

### Strengths and Limitations

Ideally, the impact of health care interventions should be assessed using an experimental study design, such as a randomised controlled trial [17]. However, where interventions are implemented across an entire population, researchers do not have control over intervention allocation, so nonexperimental study designs must be used instead [17,18]. Interrupted time-series methods have several advantages over other quasi-experimental studies, because they are less likely to be influenced by a number of biases [18]. For example, underlying increasing or decreasing secular trends may contribute to observed intervention effects. This variation may also occur where cyclical effects introduce localised upward or downward trends in the time series. Interventions that may be short-lived may erroneously report maximal effects if short time series are analysed. Finally, autocorrelated data means that adjacent data points can be more similar (positive autocorrelation) or dissimilar (negative autocorrelation), leading to under- or overestimates of effect, respectively.

However, as with all nonexperimental designs, causal inference from interrupted time-series designs is limited because it is impossible to rule out alternative explanations for observed changes in time series [18]. One such explanation is a change in the way data are recorded (data artefact), and in our study, coding of deaths changed from International Classification of Diseases-9 to International Classification of Diseases-10 in 2001. However, we think this is an unlikely explanation for our findings: a bridge coding study showed no impact on the number of drug-poisoning deaths [19], and the ONS database does not use International Classification of Diseases codes to identify which drugs were involved [10]. Furthermore, as the coroner's primary function is to investigate the possibility of criminal involvement, information from death certificates may not be ideal for surveillance of drug-related deaths [10]. Information on the death certificate may be incomplete, as toxicological examination may not be conducted or may only test for a limited range of drugs; about 10% of drug-poisoning deaths have no specific information on the drug(s) taken [4]. Where multiple substances are mentioned on the death certificate, it is not possible to establish which drug was the most likely to have caused the death. This may lead to misclassification of some deaths as due to paracetamol. However, there is no evidence to suggest that the recording practice of coroners has changed over time, and no guidelines or recommendations about the investigation of poisoning deaths have been published that may have changed their recording with the ONS system.

An alternative explanation is that an abrupt change in the population at risk coincided with the introduction of the intervention. This change could occur if, for example, the 1998 regulations inadvertently encouraged retailers to be more cautious about selling paracetamol to young girls, the group most frequently admitted to hospital for paracetamol poisoning [8]. Again, we think this is unlikely because age-specific mortality rates were similar across all age groups during the time series.

A third explanation is that external influences may have caused longer-term cyclical trends that we have not taken into account, such as changing unemployment or other macrolevel effects [20]. Such influences could explain the similarity of the three drug-poisoning time series in our study.

A major limitation of previous studies assessing the impact of the 1998 regulations on paracetamol-poisoning mortality is that no comparison group was used. However, identifying a suitable comparator is not straightforward, and hence we chose multiple comparison groups. Aspirin share many of the characteristics of paracetamol: it is a readily available analgesic and is freely available over the counter without prescription. However, aspirin is not an ideal comparator as it is less toxic than paracetamol, many more deaths involving aspirin also involve other drugs, the median age at death is greater, and fewer aspirin-related deaths are considered suicides. Furthermore, aspirin was also subject to pack-size restrictions, which may explain why trends in aspirin-related deaths are similar to paracetamol. Antidepressant-related deaths are far more similar to paracetamol in terms of median age at death and involvement in suicide, however, they too are frequently taken with other drugs and unlike paracetamol, are only available in prescription. Given these differences, it is even more striking that mortality trends involving these two drugs are similar. Paracetamol compounds include a number of different paracetamol-containing drugs. Like antidepressants, many are only available on prescription, and some are far more toxic in overdose than paracetamol. One such compound, co-proxamol, was recently banned in the United Kingdom because of its high toxicity and frequent involvement in poisoning deaths [21].

### Conclusions

We have shown that paracetamol-poisoning deaths decreased around the same time that the regulations were introduced. This observation is consistent with other studies [8,9,22]. However, we have also shown that poisoning deaths involving aspirin, antidepressants, and to a lesser degree, paracetamol compounds followed similar trends. This finding raises the question whether the concurrent introduction of the 1998 regulations and reduction in paracetamol-poisoning deaths following was coincidental rather than causal. We found little evidence to support the hypothesis that the 1998 regulations limiting pack size resulted in a greater reduction in poisoning deaths than occurred for other drugs or nondrug poisoning suicides.

## Abbreviations

ONS: Office for National Statistics

## References

[pmed-0040105-b001] Gunnell D, Murray V, Hawton K (2000). Use of paracetamol (acetaminophen) for suicide and nonfatal poisoning: Worldwide patterns of use and misuse. Suicide Life Threat Behav.

[pmed-0040105-b002] Davern TJ, James LP, Hinson JA, Polson J, Larson AM (2006). Measurement of serum acetaminophen-protein adducts in patients with acute liver failure. Gastroenterology.

[pmed-0040105-b003] Larson AM, Polson J, Fontana RJ, Davern TJ, Lalani E (2005). Acetaminophen-induced acute liver failure: Results of a United States multicenter, prospective study. Hepatology.

[pmed-0040105-b004] Office for National Statistics (2006). Report: Deaths related to drug poisoning: England and Wales, 2000–2004. Health Stat Q.

[pmed-0040105-b005] Committee on Safety of Medicines and the Medicines Control Agency (1997). Paracetamol and aspirin. Current Problems in Pharmacovigilance.

[pmed-0040105-b006] Medicines Control Agency, Department of Health (1996 November 22). Analgesic medicines available without prescription: Proposed changes to product information and sale or supply of paracetamol.

[pmed-0040105-b007] Morgan O, Majeed A (2005). Restricting paracetamol in the UK to reduce poisoning: A systematic review. J Public Health (Oxf).

[pmed-0040105-b008] Morgan O, Griffiths C, Majeed A (2005). Impact of paracetamol pack size restrictions on poisoning from paracetamol in England and Wales: An observational study. J Public Health (Oxf).

[pmed-0040105-b009] Hawton K, Simkin S, Deeks J, Cooper J, Johnston A (2004). UK legislation on analgesic packs: Before and after study of long term effect on poisonings. BMJ.

[pmed-0040105-b010] Christophersen O, Rooney C, Kelly S (1998). Drug-related mortality: Methods and trends. Popul Trends.

[pmed-0040105-b011] Brock A, Griffiths C (2001). Trends in suicide by method in England and Wales 1979 to 2001. Health Stat Q.

[pmed-0040105-b012] Office for National Statistics (2002). Deaths related to drug poisoning: Results for England and Wales, 1993–2000. Health Stat Q.

[pmed-0040105-b013] Wagner AK, Soumerai SB, Zhang F, Ross-Degnan D (2002). Segmented regression analysis of interrupted time series studies in medication use research. J Clin Pharm Ther.

[pmed-0040105-b014] Chatfield C (1989). The analysis of time series. An introduction. 4th edition.

[pmed-0040105-b015] Altman D (1999). Practical statistics for medical research.

[pmed-0040105-b016] McDowall D, McCleary R, Meidinger E, Hay R (1980). Interrupted time series analysis.

[pmed-0040105-b017] Eccles M, Grimshaw J, Campbell M, Ramsay C (2003). Research designs for studies evaluating the effectiveness of change and improvement strategies. Qual Saf Health Care.

[pmed-0040105-b018] Cook T, Campbell D (1979). Quasi-experimentation. Design and analysis issues for field settings.

[pmed-0040105-b019] Griffiths C, Rooney C (2003). The effect of the introduction of ICD-10 on trends in mortality from injury and poisoning in England and Wales. Health Stat Q.

[pmed-0040105-b020] Lester D, Yang B (2003). Unemployment and suicidal behaviour. J Epidemiol Community Health.

[pmed-0040105-b021] Hawton K, Simkin S, Deeks J (2003). Co-proxamol and suicide: A study of national mortality statistics and local non-fatal self poisonings. BMJ.

[pmed-0040105-b022] Hawton K, Townsend E, Deeks J, Appleby L, Gunnell D (2001). Effects of legislation restricting pack sizes of paracetamol and salicylate on self poisoning in the United Kingdom: Before and after study. BMJ.

